# Antisynthetase Syndrome Masquerading As Psoriasis and Psoriatic Arthritis: A Case Report

**DOI:** 10.7759/cureus.90904

**Published:** 2025-08-24

**Authors:** Eunice Y Chow, Kim H Tran, Raymond Lai

**Affiliations:** 1 Medicine/Dermatology, University of Alberta, Edmonton, CAN; 2 Medicine and Dentistry, University of Alberta, Edmonton, CAN; 3 Laboratory Medicine and Pathology, University of Alberta, Edmonton, CAN

**Keywords:** antisynthetase syndrome, cutaneous manifestations, dermatologic, dermatomyositis, psoriasis

## Abstract

Antisynthetase syndrome (ASyS) is a subtype of idiopathic inflammatory myopathy. It may have cutaneous findings like psoriasis and dermatomyositis, making clinical diagnosis challenging. We report a case of a 49-year-old woman who presented with initial symptoms of psoriasis and psoriatic arthritis: scaly patches on the scalp, scaly patches on the elbows, hyperkeratotic scaly plaques on her palms, and inflammatory joint symptoms. However, the development of phototherapy-induced flares and resistance to topical steroids prompted reconsideration of her diagnosis. Further investigations revealed the presence of anti-Jo-1 antibodies, which led to the diagnosis of ASyS. This case emphasizes the need for a broad differential diagnosis when evaluating psoriasiform skin changes, particularly in cases with atypical features or resistance to standard treatments.

## Introduction

Antisynthetase syndrome (ASyS) is a subtype of idiopathic inflammatory myopathy (IIM), a group of autoimmune diseases characterized by muscle weakness and inflammation, with extra-muscular involvement [1,2]. ASyS patients often have autoantibodies against their aminoacyl-tRNA synthetases (ARSs), with anti-Jo-1 (antibodies against histidyl) being the most common [2]. The cardinal features of ASyS include arthritis, fever, interstitial lung disease (ILD), mechanic’s hands, Raynaud’s phenomenon, and myositis, with arthritis, ILD, and myositis being the most common triad of symptoms at initial presentation [2].

The most recognized skin finding for ASyS is mechanic’s hands, which is characterized by hyperkeratotic, scaly, fissured plaques on the fingers/palms, especially the lateral aspect of the digits [3]. However, mechanics’ hands have a low sensitivity and can often be misdiagnosed as hand dermatitis or variants of psoriasis [3].

Psoriasis, on the other hand, is a chronic inflammatory condition characterized by localized or widely distributed scaly patches or plaques that can be accompanied by inflammatory joint findings (i.e., psoriatic arthritis). Meanwhile, dermatomyositis is an IIM characterized by distinct skin lesions and weakness in the proximal muscles, with possible accompanying polyarthritis/arthralgias and visceral organ involvement [4]. Both dermatomyositis and psoriasis have cutaneous and musculoskeletal features that may be similar to ASyS, making it difficult to clinically differentiate these conditions without appropriate histological and serological investigations.

We report on a patient with initial clinical findings most consistent with psoriasis and psoriatic arthritis, whose eventual diagnosis turned out to be ASyS.

## Case presentation

**Figure 1:**
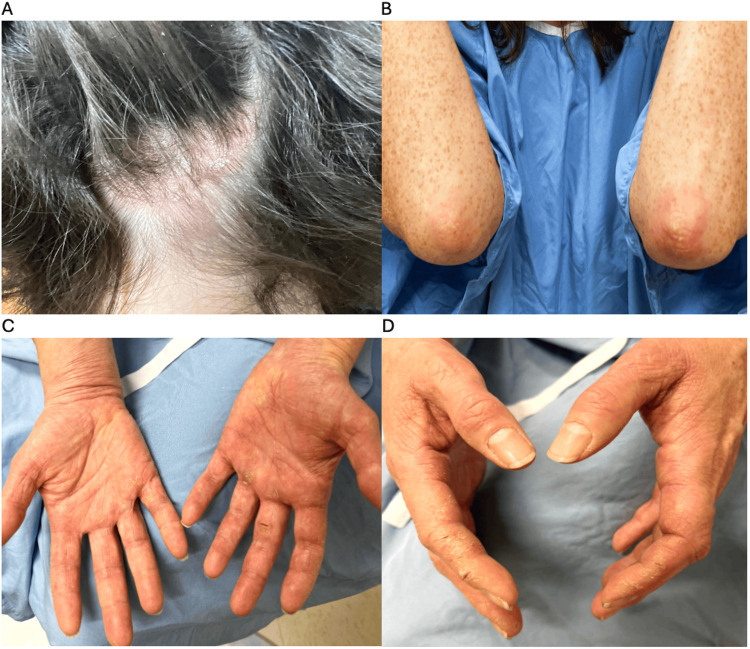
Clinical presentation of the patient's scalp, elbow, and hands before treatment (A) Red, scaly patches on the occipital scalp. (B) Red, scaly patches on the elbows. (C) Hyperkeratotic scaly papules and plaques with a fissure on the volar surfaces of the hands and fingers (specifically over the volar side of the metacarpophalangeal joints and ventral surfaces of the proximal phalanges). (D) Scaly, fissured plaques on the lateral aspects of the second digits bilaterally.

A 49-year-old female was seen for an itchy, scaly rash on the occipital scalp that started eight months previously. She reported fatigue and malaise with pain and swelling in various joints. She would experience joint stiffness in her spine and hands in the morning, lasting 30-60 minutes. Her past medical history included endometriosis, asthma, hypertension, and a maternal history of psoriasis. Her medications included fluticasone, salbutamol, perindopril, progesterone, and betamethasone/calcipotriol gel. On examination, there were well-demarcated, erythematous, scaly patches on her occipital scalp and scaly patches on the elbows (Figure 1A-1B). There were hyperkeratotic scaly papules and plaques on the palms and the lateral aspects of the index fingers and medial aspects of the thumbs (Figure 1C-1D).

The fungal scraping of the palms for dermatophyte infection was negative. Skin biopsies of the right-hand second digit and left elbow were performed. The histopathology report revealed psoriasiform epidermal hyperplasia, mild spongiosis, and patchy lymphocytic exocytosis. Fungal stains were negative, and interface changes were not seen. In the papillary dermis, there was a sparse superficial lymphocytic infiltrate; eosinophils were absent. The mid and deep dermis were unremarkable; no evidence of mucin deposition in the dermis was detected.

Based on the clinical features and histological findings, a diagnosis of psoriasis was rendered. Treatments included betamethasone/calcipotriol gel for her scalp, elbows, and hands. On follow-up, while her scalp improved, her elbow and hand eruptions did not. She was sent to do narrow-band ultraviolet B phototherapy for her hands and was referred to rheumatology for her joint symptoms.

**Figure 2:**
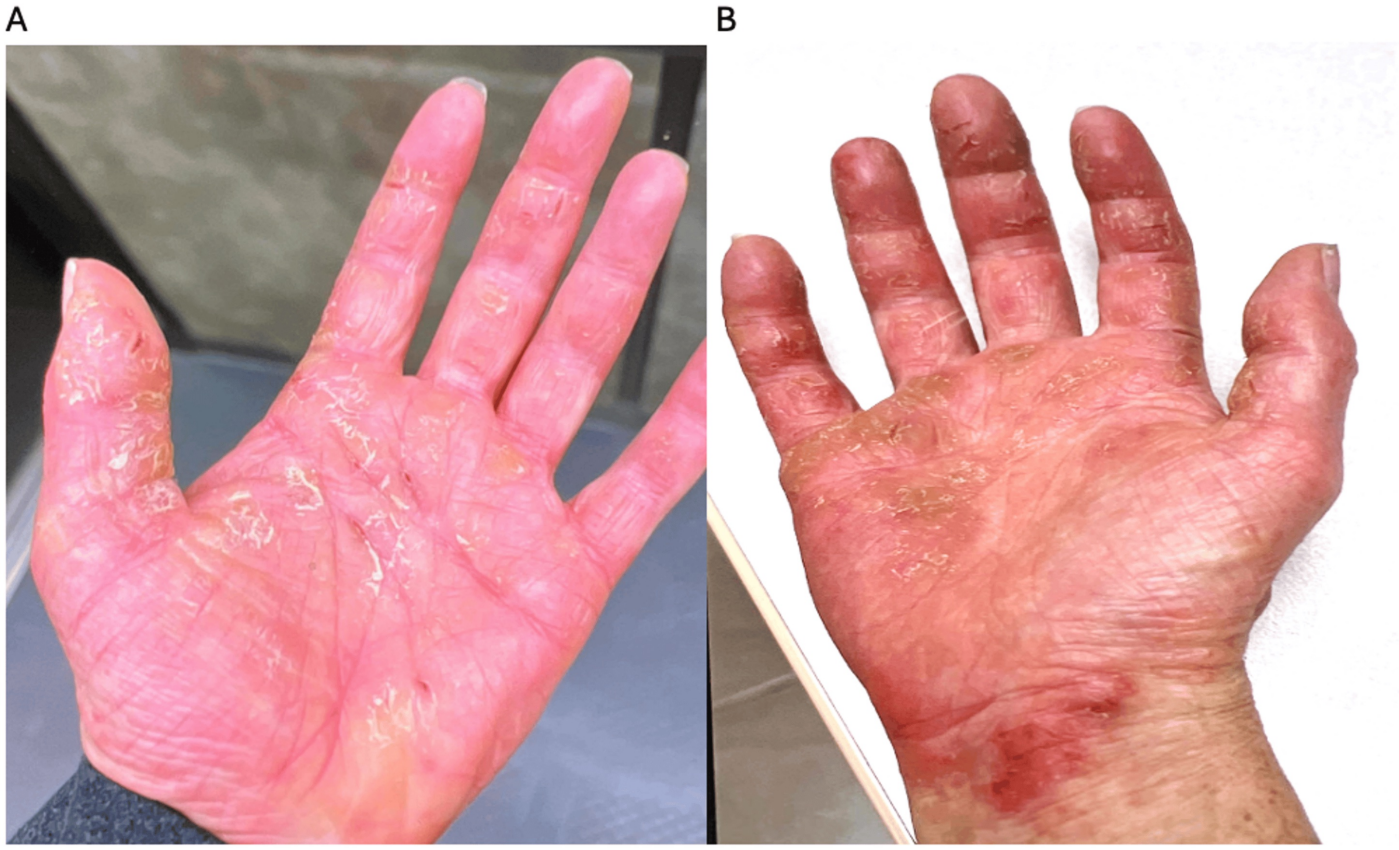
Clinical presentation of the patient's hands after phototherapy (A) Inflamed red scaly keratotic papules and plaques with fissures over the left palm. (B) Inflamed, red scaly keratotic papules and plaques with fissures over the right palm, with involvement of the volar wrist.

The patient had approximately three months of phototherapy for her hands, but during that time, the eruption on her hands worsened, becoming red and sunburn-like (Figure 2A-2B). Rheumatology found signs and symptoms of inflammatory joint disease and diagnosed her with psoriatic arthritis based on her concomitant diagnosis of psoriasis.

**Figure 3:**
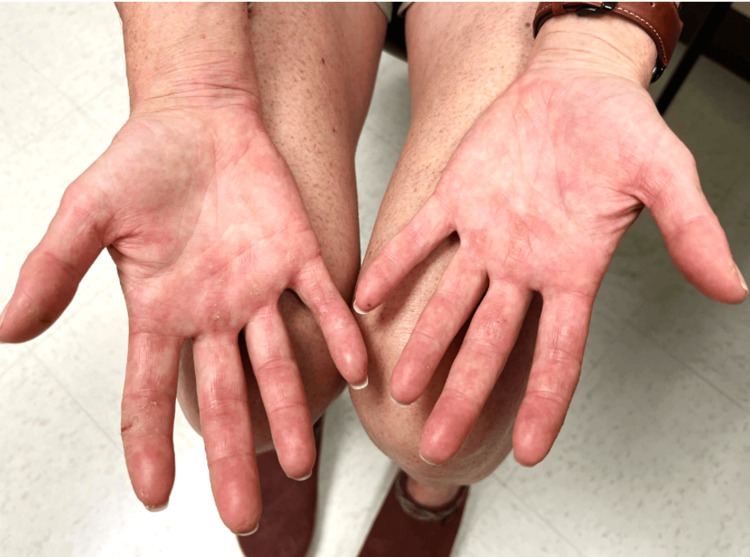
Clinical presentation of the patient's hands after initiation of systemic therapy. A few residual scaly erythematous papules on the bilateral palms a few weeks after initiation of methotrexate 20 mg/week.

With the initiation of methotrexate (20 mg/week), she noticed improvement in her skin and joint symptoms approximately three months after starting treatment (Figure 3). After her skin flared with phototherapy, the diagnosis of psoriasis was reconsidered.

Additional investigations revealed positive anti-Jo-1 antibodies but negative antinuclear antibodies and extractable nuclear antibodies. X-rays of the sacroiliac joint, hand, wrist, and ankles were normal. There were no other abnormal laboratory results. At this point, the diagnoses considered included dermatomyositis or ASyS. Upon subsequent review by her rheumatologist, he agreed that the patient’s clinical findings and investigations may be most consistent with ASyS. Subsequent investigations revealed normal pulmonary function tests and no signs of ILD or malignancies.

**Figure 4:**
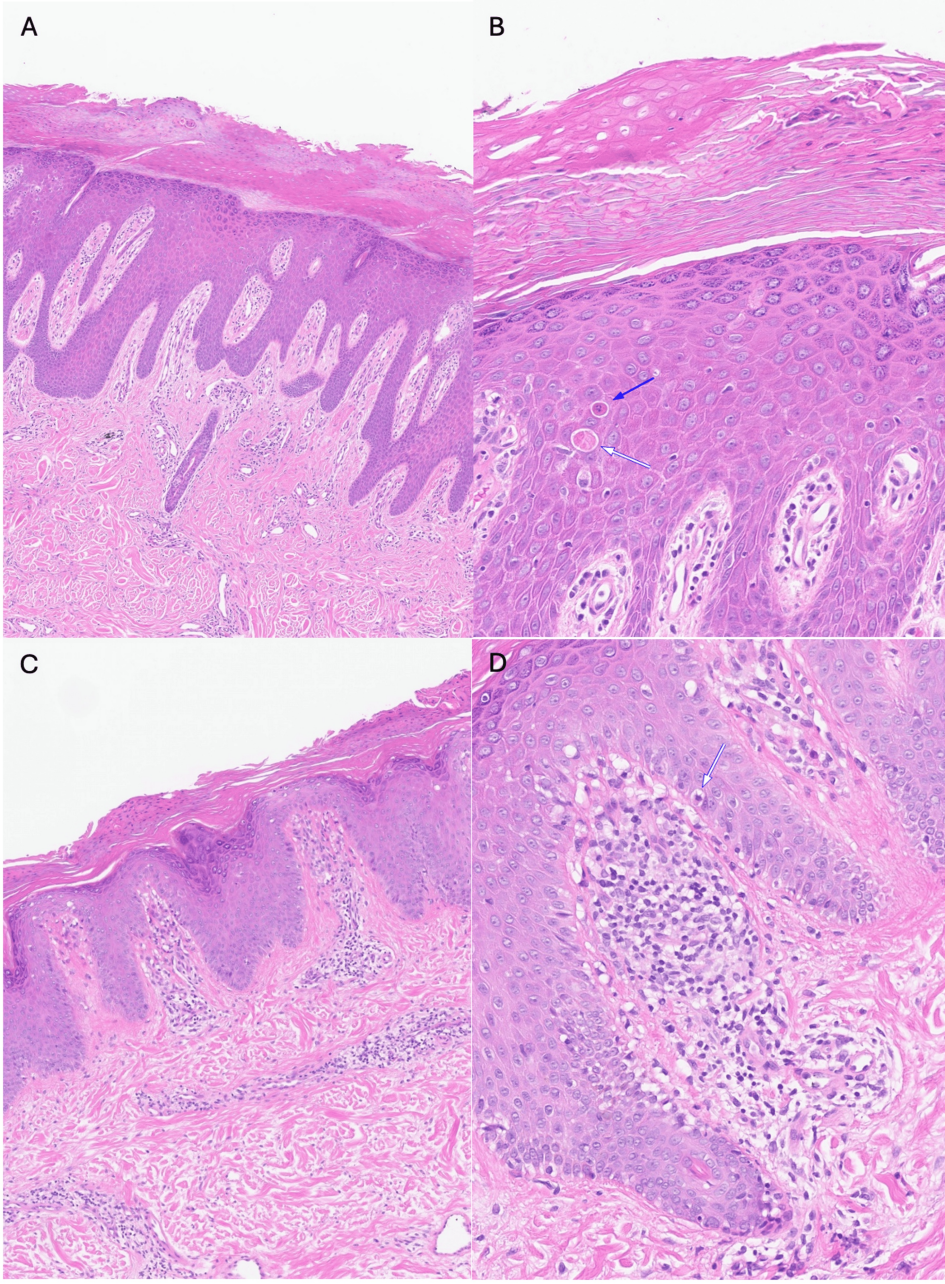
Histological features of the skin biopsy from the right-hand second digit (A & B) and the left elbow (C & D) (A, C) On low magnification, the skin biopsy showed irregular psoriasiform hyperplasia as well as parakeratosis alternating with orthokeratosis. (B, D) On high magnification, areas of parakeratosis and orthokeratosis were noted. Dyskeratotic cells (white and blue arrows) were identified within the epidermis, and the granular layer is preserved. Spongiosis/lymphocytic exocytosis was minimal. Suprapapillary thinning or dilated blood vessels was not a feature.

Considering these new clinical findings, the skin biopsies from the right-hand second digit and left elbow were re-examined. Microscopic examination of the second digit revealed irregular psoriasiform epidermal hyperplasia (Figure 4A-4B). While features such as the preservation of the granular layer, the absence of neutrophilic debris in the corneal layer, and the lack of supra-papillary thinning/dilated blood vessels in the papillary dermis may be compatible with partially treated psoriasis, the presence of dyskeratotic cells and alternating parakeratosis/orthokeratosis vertically and horizontally was more supportive of a diagnosis of mechanic’s hands. Microscopic examination of the left elbow showed irregular psoriasiform epidermal hyperplasia and patchy parakeratosis (Figure 4C-4D). Focally, vacuolation of the basal epidermal cells with blurring of the epidermal/dermal junction and rare dyskeratotic cells were identified, suggestive of interface changes, which may be pathogenetically related to the underlying connective tissue disorder.

## Discussion

In this case, the patient presented with scaly red patches/plaques on the scalp, elbows, and hands accompanied by inflammatory joint symptoms. These findings initially suggested a diagnosis of psoriasis with psoriatic arthritis, further supported by the histopathologic finding of psoriasiform epidermal hyperplasia. However, the development of phototherapy-induced worsening of her eruption prompted reconsideration. In the setting of a potential diagnosis of dermatomyositis, the patient’s findings may be interpreted as Gottron’s sign (mildly scaly red patches over the elbows), inverse Gottron’s papules (scaly red papules over the volar metacarpophalangeal joint of the hands), scalp involvement of dermatomyositis, mechanic's hands, and muscle/joint involvement [5].

However, the detection of anti-Jo-1 antibodies ultimately led to the diagnosis of ASyS. Hum et al. emphasized that ASyS can present with dermatomyositis-like rashes, complicating its classification [6]. The European Neuromuscular Centre recommended classifying patients with ARS antibodies and dermatomyositis-like rashes as having ASyS with dermatomyositis-like rash rather than dermatomyositis. In a registry of 1,054 patients, 28% of ASyS patients exhibited dermatomyositis-specific rashes without an increased malignancy risk, distinguishing them from dermatomyositis patients [6]. Mechanic's hands are more commonly observed in ASyS patients with dermatomyositis-like rashes compared to those with dermatomyositis alone, suggesting that the presence of mechanic's hands may favor an ASyS diagnosis [6]. Additionally, hallmark dermatomyositis rashes such as heliotrope rash, V-neck sign, violaceous rash, and shawl sign are rare in ASyS, aiding in the differentiation of these conditions [6].

In this case, the finding of anti-Jo-1 antibody, the absence of hallmark dermatomyositis rashes, and the presence of mechanic's hands supported the ASyS diagnosis. The phototherapy-induced flares of her hand rash prompted reconsideration of her diagnosis. While photosensitivity is well-documented in dermatomyositis, it is rarely reported in ASyS. However, Chan et al. highlighted its presence in ASyS as part of the broader spectrum of skin manifestations [3]. The patient’s non-pruritic hand dermatitis, which did not improve with topical steroids, aligns with the classic description of mechanic’s hands [7].

This case has exemplified the challenge in diagnosing mechanic's hands histologically, since one of its key features, namely psoriasiform hyperplasia, is shared by partially treated psoriasis and chronic/partially treated spongiotic dermatitis. Nonetheless, certain histological clues favor the diagnosis of mechanic’s hands, including the presence of dyskeratotic cells (colloid bodies), interface changes (i.e., vacuolation of the basal epidermal layer), and mucin deposits in the dermis [8]. More recent publications have also highlighted the pseudo-checkerboard pattern, with parakeratosis alternating with orthokeratosis horizontally and vertically [9]. This feature was identified in the skin biopsy from the second digit of the right hand on re-examination. This feature may be subtle, and its detection requires a high level of suspicion.

## Conclusions

This case highlights the diagnostic challenges posed by ASyS, which can mimic both psoriasis and dermatomyositis clinically and histopathologically. It underscores the importance of differentiating psoriasis from autoimmune myositis conditions such as ASyS and dermatomyositis, given the distinct management and screening protocols required. Early recognition of ASyS is crucial due to its high risk of ILD, necessitating timely detection and intervention. Furthermore, distinguishing dermatomyositis from ASyS is essential, as dermatomyositis warrants malignancy screening due to its cancer risk, while ASyS necessitates vigilant pulmonary monitoring.

## References

[1] Cox JT, Gullotti DM, Mecoli CA (2017). "Hiker's feet": a novel cutaneous finding in the inflammatory myopathies. Clin Rheumatol.

[2] Huang K, Aggarwal R (2020). Antisynthetase syndrome: a distinct disease spectrum. J Scleroderma Relat Disord.

[3] Chan AR, Osman M, Yacyshyn E, Gniadecki R (2024). Cutaneous manifestations of anti-synthetase syndrome: case series and literature review. JEADV Clin Pract.

[4] Chu D, Yang W, Niu J (2024). Concurrence of dermatomyositis and psoriasis: a case report and literature review. Front Immunol.

[5] DeWane ME, Waldman R, Lu J (2020). Dermatomyositis: clinical features and pathogenesis. J Am Acad Dermatol.

[6] Hum RM, Lilleker JB, Lamb JA (2024). Comparison of clinical features between patients with anti-synthetase syndrome and dermatomyositis: results from the MYONET registry. Rheumatology (Oxford).

[7] Concha JS, Merola JF, Fiorentino D, Werth VP (2018). Re-examining mechanic's hands as a characteristic skin finding in dermatomyositis. J Am Acad Dermatol.

[8] Mii S, Kobayashi R, Nakano T (2009). A histopathologic study of mechanic's hands associated with dermatomyositis: a report of five cases. Int J Dermatol.

[9] Oteiza Rius I, Antoñanzas Pérez J, Morelló Vicente A, España A (2024). Mechanic's hands: clinical and histopathologic findings. Actas Dermosifiliogr.

